# Cardioprotective SUR2A promotes stem cell properties of cardiomyocytes^☆^^☆☆^

**DOI:** 10.1016/j.ijcard.2013.07.182

**Published:** 2013-10-12

**Authors:** Stephen C. Land, David J. Walker, Qingyou Du, Aleksandar Jovanović

**Affiliations:** Medical Research Institute, Division of Cardiovascular and Diabetic Medicine, Ninewells Hospital & Medical School, University of Dundee, Dundee DD1 9SY, UK

**Keywords:** SUR2A, Cardioprotection, Stem cells

Development of stem cell therapies to treat cardiovascular disease is limited by the ability to regulate stem cell differentiation and by poor cell survival once grafted to sites of damage in the heart [1]. SUR2A belongs to a group of “atypical” ABC proteins as, although possessing a structure of an ABC protein, it does not seem to mediate transport. Instead, SUR2A binds to inward rectifier Kir6.2 to form cardiac sarcolemmal ATP-sensitive K^+^ channels. The binding of SUR2A to Kir6.2 serves a dual purpose: 1) it allows translocation of the channel to the sarcolemma and 2) contributes to the channel regulation (reviewed in ref. [2]). *In vivo*, K_ATP_ channels exist as a multiprotein complex that, besides pore-forming Kir6.1/Kir6.2 and regulatory SUR2A subunits, also contain a string of glycolytic and ATP-producing enzymes including creatine kinase, GAPDH and M-LDH. It has been shown that the changes in levels of SUR2A alone have a profound effect on myocardial susceptibility to different types of metabolic stresses including hypoxia, ischemia, ischemia-reperfusion and stimulation with β-adrenergic agonists (reviewed in ref. [3]). Increase in intracellular SUR2A raises the number of fully-assembled cardioprotective K_ATP_ channels resulting in 1) earlier opening of K_ATP_ channels in response to stress and 2) increased subsarcolemmal ATP due to increased recruitment of creatine kinase and glycolytic enzymes to the K_ATP_ channel protein complex. Improved timing of K_ATP_ channel opening as well as increased subsarcolemmal production of ATP seems to mediate cardioprotection afforded by SUR2A [3]. The efficacy and safety of SUR2A led to suggestion that manipulation of its expression in cardiac tissue could be a promising therapeutic strategy against ischemic heart disease [3]. On the other hand, it has been also shown that cardiac stem cells regenerate infarcted myocardium and improve cardiac function [1]. Whether SUR2A-based therapeutic strategy of heart ischemia is compatible/complementary with stem cell therapy is at the present unknown. To assess possible relationship between SUR2A and cardiac stem cells, we have collected mouse fetal hearts at E12.5 stage (for details of heart harvesting at this stage see ref. [4]) and infected them with adenovirus containing SUR2A (AV-SUR2A) or luciferase (control; method of infection is described in ref. [5]). Hearts were used 24 h later for biochemical assessments. Infection of the heart with AV-SUR2A increased SUR2A mRNA levels for ~ 10 times showing that infection was sufficient to produce significant increase in SUR2A (Fig. 1). At the same time, expression of genes indicating differentiation of cardiomyocytes, as measured by quantitative real time RT-PCR (for detailed methodology see ref. [5]), was dramatically decreased; troponin C for ~ 30 times and GATA 4 for ~ 4 times. BMZ, a housekeeping gene, was not affected at all (Fig. 1). These findings suggest that SUR2A shifted embryonic cardiomyocytes towards less differentiated state. It is well established that ERK1/2 pathway is responsible for heart embryonic development (reviewed in ref. [6]). In order to determine whether SUR2A affects this signaling pathway we have measured phosphorylation of ERK following infection with AV-SUR2A. Using Western blotting (for details of methodology see ref. [4]), we have found significant decrease in ERK1/2 phosphorylation in hearts infected by AV-SUR2A (Fig. 2), which was associated with dramatic increase in expression of stem cell pluripotency marker mRNAs (Oct-4, Sox2 and NANOG; Fig. 2) whose abundance is known to promote non-differentiating, proliferative growth in cardiac and other tissues [7]. It is well established that pluripotent stem cells can be generated from somatic cells by expression of reprograming factors, such as Oct-4 and Sox2. It has been proposed that it's the ratio of these genes to one another that really matters in reprogramming [8]. Here we have found that although SUR2A increased the expression of all 3 genes, the effect was greatest for Sox2 and Oct-4 strongly suggesting that cardiomyocytes were indeed reprogrammed into stem cells. Ratio of Sox2/Oct-4/NANOG seems to be a hallmark of cardiomyocyte non-differentiation and recent demonstration that NANOG over-expression raises Oct-4 and Sox2 and hinders cardiomyocyte differentiation strongly supports our data [9]. These results suggest that SUR2A “tip” cardiomyocytes towards a primed state capable of non-differentiating growth which is characteristic to cardiomyocyte precursor cells. When considering that, in addition to findings from this study, increased SUR2A levels are also efficient in protecting both embryonic and adult heart cells against severe metabolic stress (including ischemia; refs. [3,5]), this protein seems perfect to be used as a tool to 1) keep stem cells into non-differentiated state, while 2) increasing their resistance to metabolic stress. Stem cells overexpressing SUR2A would be easier to maintain in non-differentiated state, while such cells would survive better when they are grafted to treat ischemia/myocardial infarction. As methodologies securing long-lasting expression of a gene are now well developed [10], there are no any technical obstacles in obtaining cardiac stem cells overexpressing SUR2A for therapeutic purposes. The properties of SUR2A to keep stem cells non-differentiated and to increase their resistance to metabolic stress suggest that SR2A overexpressing stem cells deserve to be seriously tested as a potential therapy against heart ischemia, including the myocardial infarction.

## Figures and Tables

**Fig. 1:**
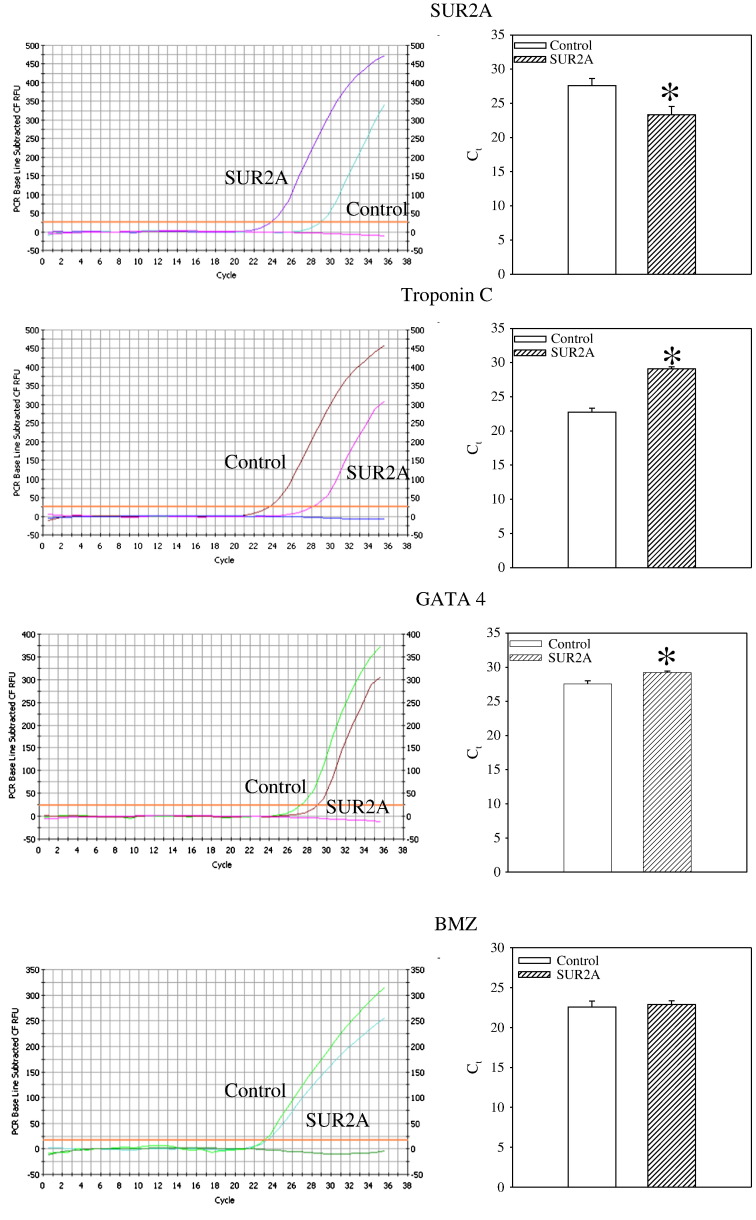
Infection of fetal heart by SUR2A results in decreased expression of cardiomyocyte expression markers. Fetal hearts were collected at E12.5 stage. Original real time RT-PCR progress curves for SUR2A, troponin C, GATA 4 and housekeeping gene BMZ and corresponding graphs depicting cycling threshold. Each bar represents mean ± S.E.M. (n = 3). *P < 0.05. Infection of heart with SUR2A increased SUR2A mRNA levels for ~ 10 times and decreased troponin C and GATA 4 mRNA for ~ 30 and ~ 4 times. Infection with SUR2A did not affect BMZ at all.

**Fig. 2:**
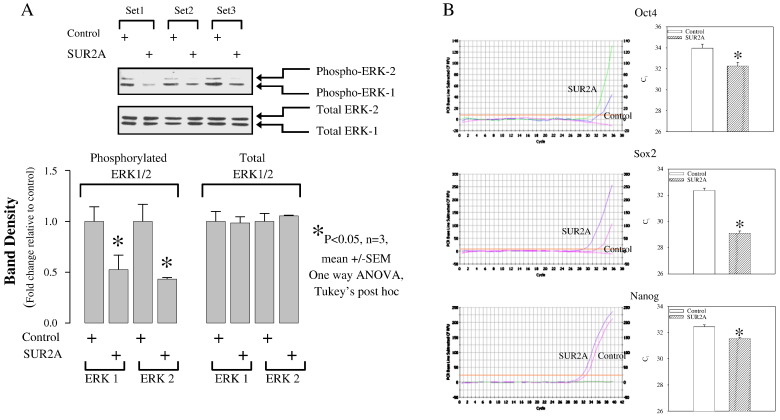
Infection of fetal heart by SUR2A results in suppressed ERK activity and heart shifted towards less differentiated state. Fetal hearts were collected at E12.5 stage. A. Original Western blotting and corresponding graphs of fetal hearts infected with luciferase (control; luciferase was used as a control as we have determined that infection with luciferase does not affect phospho- or total ERK1/2) and SUR2A. While total ERK1/2 was not affected by SUR2A, phosphorylation of ERK1/2 was. Each bar represents mean ± S.E.M. (n = 3; n is defined as the number of infected fetal hearts). *P < 0.05. B. Original real time RT-PCR progress curves for Oct4, Sox 2 and nanog and corresponding graphs depicting cycling threshold. Each bar represents mean ± S.E.M. (n = 3). *P < 0.05.
